# Supplementary material for: Elucidating the active phases of CoOx films on Au(111) in the CO oxidation reaction
Source: Nat Commun. 2023 Oct 28;14:6889. doi: 10.1038/s41467-023-42301-7 (PMC10613203; doi:10.1038/s41467-023-42301-7)
Supplement: Supplementary file 1 — Supplementary Information [file 41467_2023_42301_MOESM1_ESM.pdf]

Elucidating the active phases of CoO_x films on Au(111) in the CO Oxidation Reaction

Hao Chen[1], Lorenz J. Falling[2,3], Heath Kersell[2,4], George Yan[5], Xiao Zhao[3,6], Judit Oliver Meseguer[1], Max Jaugstetter[1], Slavomir Nemsak[2,7], Adrian Hunt[8], Iradwikanari Waluyo[8], Hirohito Ogasawara[9], Alexis T. Bell[1,10], Philippe Sautet[5,11], and Miquel Salmeron[1,3,6*]

[1] Chemical Sciences Division, Lawrence Berkeley National Laboratory, Berkeley, California 94720, United States;

[2] Advanced Light Source, Lawrence Berkeley National Laboratory, Berkeley, CA 94720, United States;

[3] Materials Sciences Division, Lawrence Berkeley National Laboratory, Berkeley, California 94720, United States;

[4] School of Chemical, Biological, and Environmental Engineering, Oregon State University, Corvallis, Oregon 97331, United States;

[5] Department of Chemical and Biomolecular Engineering, University of California, Los Angeles, Los Angeles, California 90095, United States;

[6] Department of Materials Science and Engineering, University of California, Berkeley, California 94720, United States;

[7] Department of Physics and Astronomy, University of California, Davis, CA 95616, United States;

[8] National Synchrotron Light Source II, Brookhaven National Laboratory, Upton, NY 11973, United States

[9] SLAC National Accelerator Laboratory, 2575 Sand Hill Road, Menlo Park, California 94025, United States;

[10] Department of Chemical and Biomolecular Engineering, University of California, Berkeley, California 94720, United States;

[11] Department of Chemistry and Biochemistry, University of California, Los Angeles, Los Angeles, California 90095, United States.

*Corresponding author. E-mail: mbsalmeron@lbl.gov

Supplemental methods, experimental and computational results

1.1 Fitting of Co 2p XP spectra for $CoO_{x\leq1}$/Au(111)

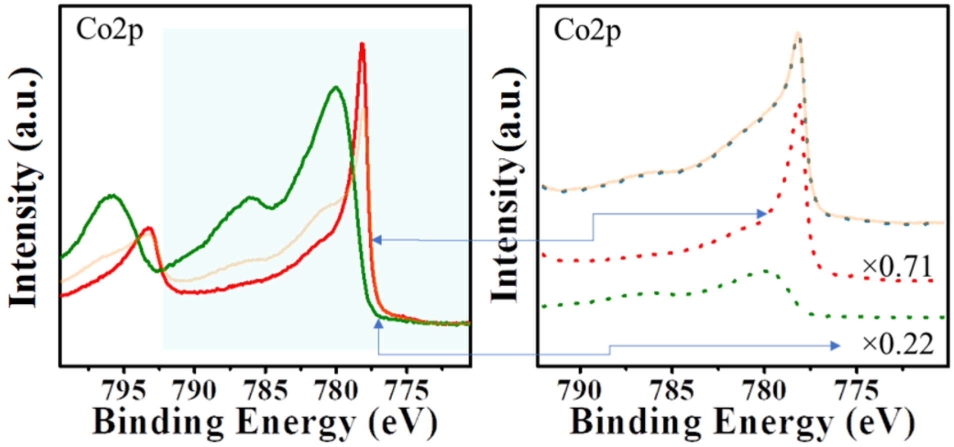

Figure S1: Linear fitting of Co2p XP spectra of partially oxidized CoO_x. (left) Red: Metallic Co (Co^0); Green: Stoichiometric CoO (Co^{2+}); Orange: partially oxidized CoO_x. Coverage: 1MLE. (Right) Linear regression analysis suggesting the ratio of Co^{2+} is 0.22 (R^2=0.973). The dotted lines in right panel correspond to the solid line in the left multiplied by the indicated factors: Co^0- 0.71(red); Co^{2+}- 0.22 (green). The sum of the dotted Co^0 and Co^{2+} curve (blue) matches well with that of the partially oxidized CoO (orange). Considering the uncertainty in the fitting process, the x was determined as 0.25. The XPS data were acquaired at beamline 23-ID-2 (IOS) of NSLS-II

1.2 Coverage of cobalt on Au from XPS, supported by SESSA simulations

<u>Metallic cobalt</u>

Due to the immiscibility of cobalt with metallic gold, cobalt will be on the surface of the Au(111) crystal used in our study. The surface spatial distribution of cobalt, however, is unknown. To approach the morphology of metallic cobalt on the surface, we used the intensity ratio of Co 3p to Au 4f (**Fig.S1a**). This ratio varies with coverage and the total amount of cobalt, or its monolayer equivalent (MLE). To showcase this, we used SESSA v2.2 [1] to simulate the Co3p / Au4f ratio with varying Co(0) coverage and amount (0.7, 0.8, 0.9, 1.0, 1.1, and 1.2 MLE). The average thickness of the cobalt islands ranged between a monolayer and a trilayer. **Figure S2** shows the workflow and the simulated intensity ratios as a function of average island thickness. As can be seen a given ratio is only linked to an island thickness for a given total amount of Co.

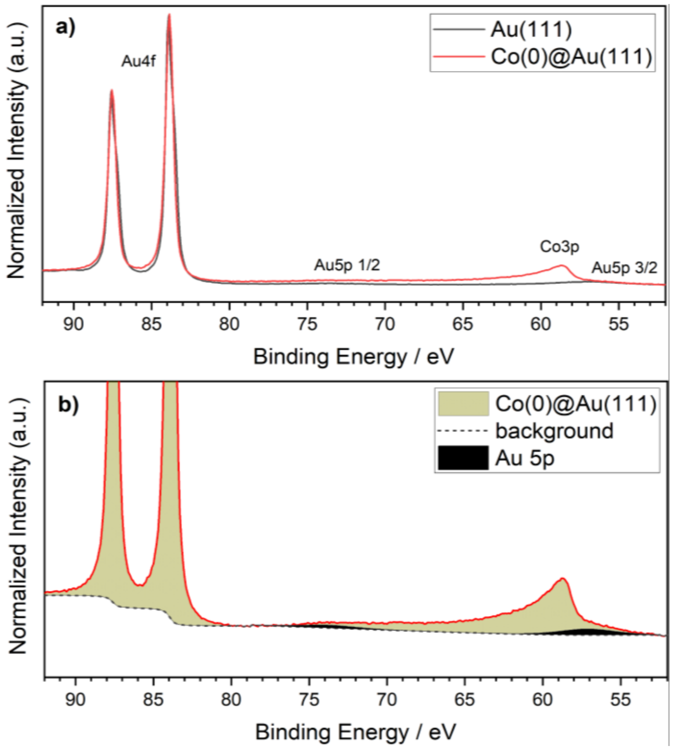

Figure S2: Au 4f, Au 5p, and Co 3p spectrum before (red) and after (black) evaporation of metallic cobalt on a Au(111) single crystal; the two spectra in a) were normalized to the background intensity at 52 eV and the Au 4f peak intensity to demonstrate the relative changes; part b) shows an example on how the peak areas were calculated; the area of Au 4f was measured above a Shirley-type background and the area of Co 3p was measured above a 2-parameter Tougaard background; the Co 3p area was corrected for the underlying Au 5p intensity, by using a fixed intensity ratio of Au 4f and Au 5p obtained from clean Au(111).

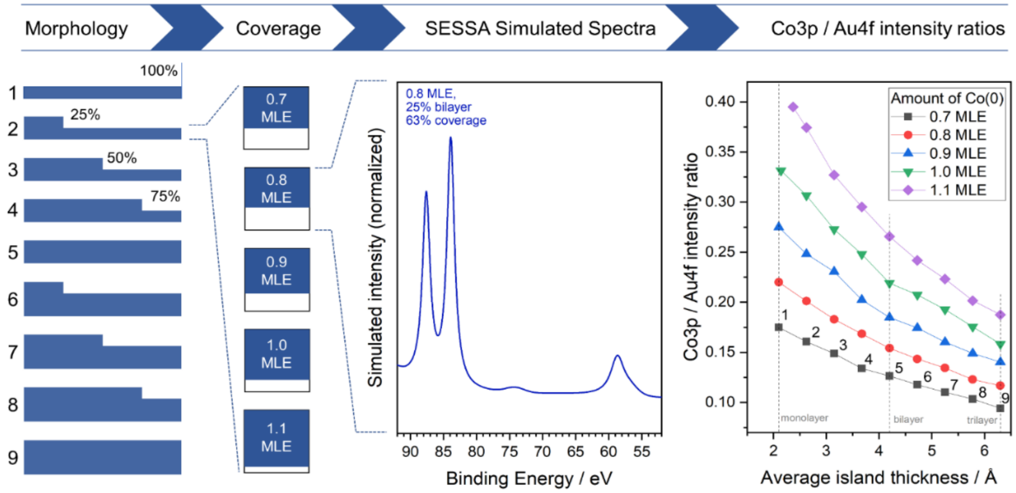

Figure S3: Workflow of the Co 3p / Au 4f intensity ratios simulated by SESSA; nine island morphologies were used as an input for SESSA; each morphology (side view on the left) results in different coverages (square top views), depending on the total amount of metallic cobalt, or monolayer equivalent (MLE); an exemplary spectrum simulated by SESSA is shown in the center; the Co 3p / Au 4f ratio of all SESSA

calculations are shown on the right; please note that all calculations used a convergence factor of $1*10^{-4}$, a Co density of 8 g/cm^3 [2]and approximated the Au 4f attenuation with an average island thickness.

To calibrate the total amount of deposited Co^0, we used a crystal quartz microbalance (QCM) at beamline 23-ID-2 (IOS) at the NSLS-II and aimed for a 1 ML of metallic cobalt, which is ~1.1 MLE on Au(111). Using the distribution of layer thicknesses from the same reference[2], i.e. 50% double layer, 35% triple layer, and 15% quadruple layer, we simulated an intensity ratio of 0.21. The measured ratio was 0.25. If we look up the simulated MLE for the measured ratio of 0.25, we obtain 1.3 MLE, which is within the error of the QCM (~20%). We can conclude that the thickness distribution of Morgenstern [3] is within the error of the QCM calibration and can serve as a model for the determination of the total amount after evaporation with the Co 3p to Au 4f intensity ratio. We did so for all measurements at beamlines 9.3.2 of the ALS and 13-2 of the SSRL. The error of this procedure is at least 20% of the MLE.

Cobalt oxides

Similar to the metal islands, the coverage of the oxidized CoO_x structures was estimated from the Co 3p to Au 4f ratio from simulations and experiments. But when transitioning from the metal to an oxide, the density drops, and the structure expands. By how much was estimated using the density and thickness of the metallic and oxide surface structures from the lattice spacing and thickness of the metallic phase [2], and the oxides [4]. Employing a layer-by-layer model for the growth of the oxide could give satisfactory agreement of the simulated and measured intensity ratios for a given MLE of Co^0. However, there were discrepancies in the data recorded at beamline 9.3.2, for which the Au 4f was attenuated more by the oxide layer than simulations suggested for a given amount of Co. The simulations suggest a larger amount of Co than was initially deposited. Inhomogeneous deposition of the metal is suspected to cause this discrepancy. The conclusions of a spread-out oxide phase and compacted islands of metallic cobalt, as well as their chemistry are unlikely to be affected by this discrepancy.

1.3 XPS quantification

For quantification of intensity changes between measurements, the spectra were normalized to their low-binding energy background. This procedure can account for the flux, the transmission function, detector efficiency, and gas phase attenuation if the sample is flat and has thin overlayers. Shirley backgrounds and peak integrations were used for quantification, unless otherwise noted.

1.4 Computed energetics of the reaction between CO and $CoO_{x\leq1}$/Au(111)

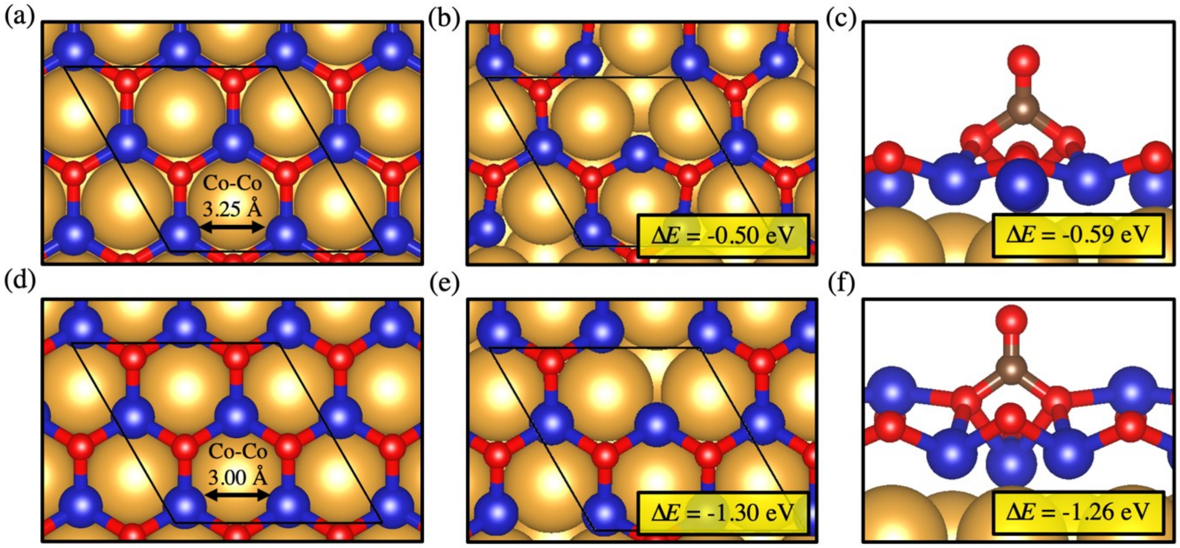

Figure S4: The electronic energy change of reacting CO with lattice O on CoO/Au(111) to form CO_2 vs. CO_3^{2-} over an epitaxial film with 3.25 Å Co-Co spacing (a), (b), and (c), and with 3.00 Å Co-Co spacing (d), (e) and (f). Although the reaction energies are similar for CO_3^{2-} and CO_2, a low partial pressure of CO_2 will thermodynamically favor the formation of CO_2 gas. Notice the more stable (-1.26 eV vs -0.59 eV) restructured film in (f) with Co atoms detached from the Au substrate.

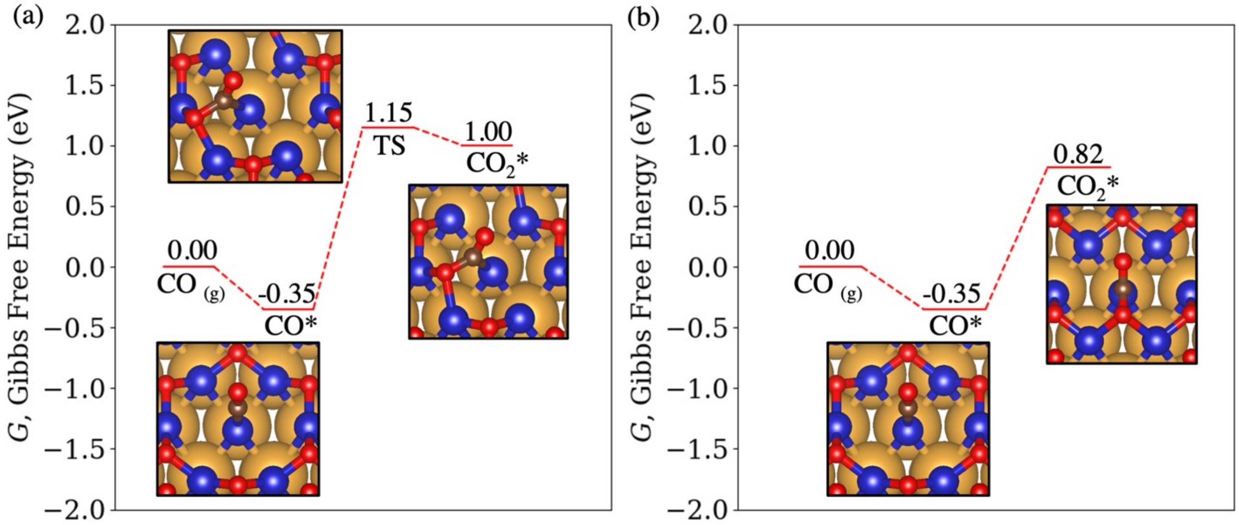

Figure S5: Free energy pathways of Co_4O_2/Au(111) reacting with CO gas. The reaction between adsorbed CO and surface O is endothermic and has a high activation barrier. (a) The adsorption of CO on exposed Co and subsequent reaction with surface O in the FCC site with respect to Co. (b) Reaction between adsorbed CO with surface O in the HCP site. The free energy of CO was calculated at 373.15 K, P_{CO} = 100 mTorr. Color scheme: Atom color: Au, yellow; C, brown; Co, blue; and O: red.

1.5 Additional XP and ResPES of CoO_x/Au

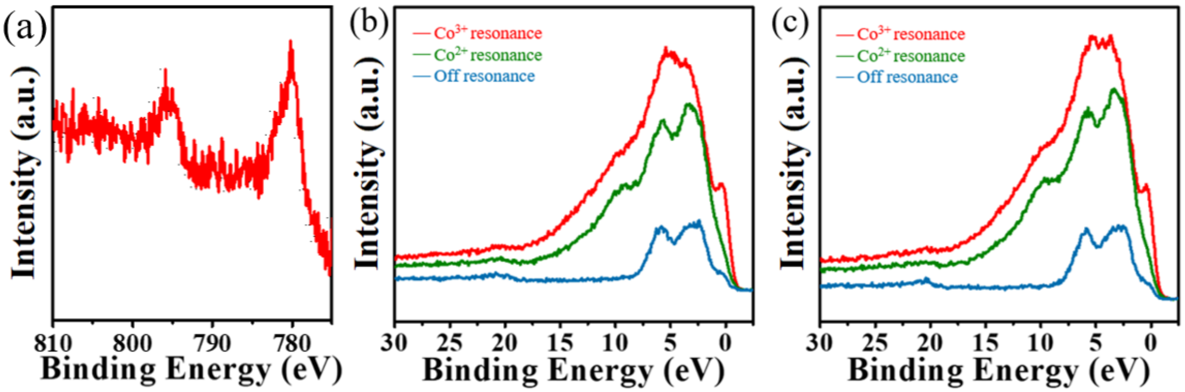

Figure S6: (a) Co2p XP spectra of 1.0 MLE reduced CoO_x film under 100mTorr O_2 at ~350 ºC; ResPES of 1.0MLE CoO_x under (b)100mtorr O_2 and (c) 100mtorr CO at RT, respectively. Data were acquaired at beamline 9.3.2 of ALS.

1.6 Description of CoO_x/Au(111) structural models used in DFT calculations

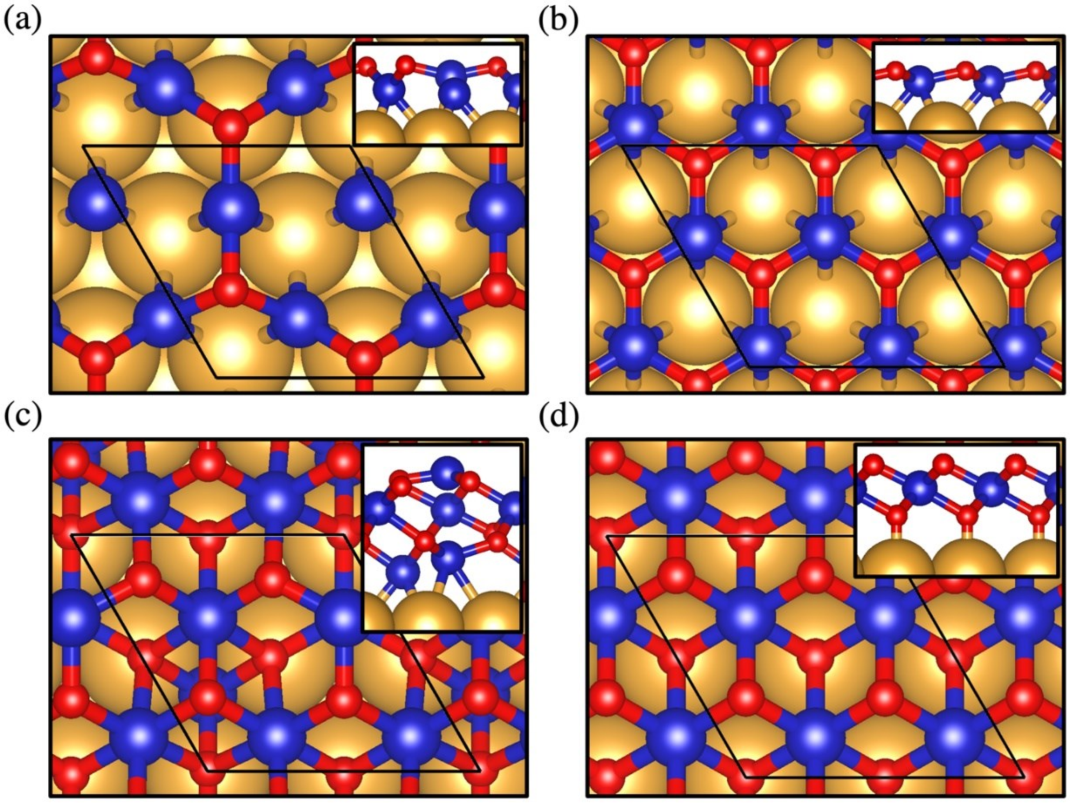

Figure S7: Structure of the CoO_x/Au(111) films to assess reactivity. (a) $CoO_{0.5}$/Au(111), (b) CoO/Au(111), (c) Co_3O_4/Au(111), (d) CoO_2/Au(111). Top views are shown in the main panels, and side views are shown in the insets. Color scheme: (Atom: color) Au: yellow; C: brown; Co: blue; O: red.

To simulate the sub-oxidized $CoO_{x<1}$/Au(111) structures (**Fig. S7a**), a (2x2) supercell of Au(111) with 4 Au layers was used as the substrate. The bottom two layers of the Au substrate were fixed to their bulk positions, while the top two layers of Au, the epitaxial $CoO_{x<1}$ layer and adsorbates were allowed to relax. Following our previous work on $CoO_{x<1}$/Pt, a row-wise antiferromagnetic structure was used for all $CoO_{x<1}$ models.[5] In the order of top-left to bottom-right in the outlined unit cell (**Fig. S7a**), the magnetic moments of the Co atoms were found to be -2.32, -1.99, 2.49, and 2.49 μ_B. For these structures, the Brillouin zone was sampled using a (7×7×1) mesh centered at the gamma point.[6]

Next, to simulate stoichiometric CoO/Au(111), a (2×2) supercell of Au(111) with 6 Au layers was used as the substrate (**Fig. S7b**). To approximate the reported nm-range order of bilayer CoO on Au(111), the lattice spacing of the epitaxial CoO film and the Au substrate were strained to 3.25 Å. The sensitivity of predicted reactivity trend was verified by changing the lattice spacing to 3.00 Å. Following reported studies, to retain the registry of the CoO film during structural optimization, all Au atoms and two interfacial Co atoms were constrained to only relax in the Z direction, while no constraint was placed on all other atoms.[7] Following previous studies of thin CoO films, a row-wise antiferromagnetic structure was used for all CoO models.[5, 8] The absolute magnetic moment of the Co cations in the films with 3.00 Å and 3.25 Å lattice spacing were found to be 2.35 and 2.57 $|\mu_B|$, respectively. For these structures, the Brillouin zone was also sampled using a (6×6×1) mesh centered at the gamma point.

As an alternative to the ¼ ML carbonate structure we had considered, we also considered the possibility of the formation of a $CoCO_3$ overlayer on Au(111). These calculations were performed over a (3×3) and a (2√3×2√3) Au(111) substrate. For these calculations, the Brillouin zone was sampled using a (5×5×1) mesh centered at the gamma point. To calculate the free energy of formation per $CoCO_3$ unit, a (1×1) Au(111) cell and a (√13×√13) CoO overlayer on (4x4) Au(111) were used as reference compounds of Au and Co. The Brillouin zone was sampled using a (15×15×1) and a (4×4×1) mesh centered at the gamma point, respectively. CO_2 gas was used as the reference compound for C.

Finally, to simulate the over-oxidized Co^{3+}-containing $CoO_{x>1}$/Au(111) structures (**Fig. S7c and S7d**), a (2×2) supercell of Au(111) with 4 Au layers was used as the substrate. The bottom two layers of the Au substrate were fixed to their bulk positions, while all other atoms were allowed to relax. Following previous studies, the occupation matrix control method was used to provide an initial guess of the spin states of the Co cations.[9] For Co_3O_4, the antiferromagnetic alignment of Co^{2+} cations in tetrahedral sites was maintained in all calculations. The magnetic moment of the outermost Co cation was found to be -2.85 μ_B, while the magnetic moments of the three Co cations in the subsurface were found to be -0.09, 1.85, and 1.84 μ_B. For CoO_2, in the order of top-left to bottom-right in the outlined unit cell, the magnetic moments of the Co atoms were found to be 0.00, 0.00, 1.26, and 1.26 μ_B (**Fig. S7d**). For these structures, the Brillouin zone was sampled using a (7×7×1) mesh centered at the gamma point.

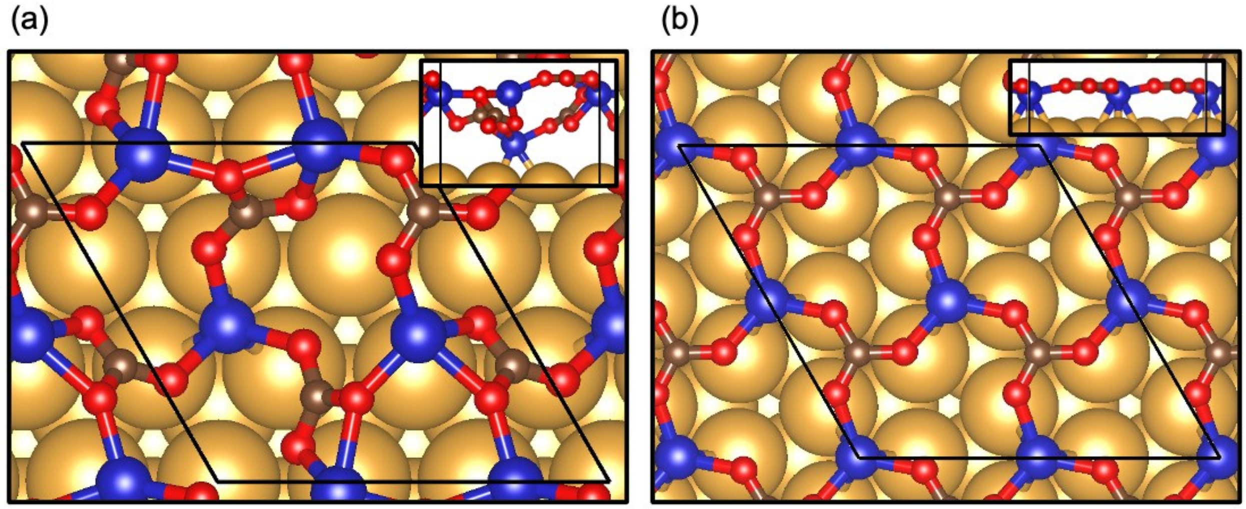

Figure S8: Structure of $CoCO_3$ overlayers on Au(111). (a) Top view of a $CoCO_3$ layer containing 4 $CoCO_3$ units on a (3×3) Au(111) substrate. The structure is unstable and dewets from the Au(111) substrate upon structural relaxation (as shown in the inset). (b) Top view of a (2×2) $CoCO_3$ layer on a (2√3×2√3) Au(111) substrate. Although an ordered structure was found, the film is very only bound to the Au substrate.

As the structure of bulk $CoCO_3$ is known, we also assessed of the stability of a $CoCO_3$ overlayer on Au(111) under a pressure of CO_2. The $CoCO_3$ structures were simulated at two coverages, as 4 $CoCO_3$ units atop a (3×3) Au(111) substrate and as 4 $CoCO_3$ units atop a (2√3×2√3) Au(111) substrate (**Figure S8**). Their optimized structures show that the $CoCO_3$ film binds weakly with the Au(111) substrate. Atop (3×3) Au(111), 3 out of 4 Co cations dewet from the substrate, while atop (2√3×2√3) Au(111), the $CoCO_3$ overlayer is loosely bound to the Au substrate, where the Co-Au distance ranges from 3.05 Å to 3.10 Å. Their stability was approximated by computing their Gibbs free energies of formation per $CoCO_3$ unit with respect to a (1×1) Au(111) cell, a (√13×√13) CoO overlayer on (4x4) Au(111) (as an approximation of CoO/Au(111) without strain on the Au lattice), and CO_2 gas at a temperature of 373 K and a partial pressure of 0.1 mTorr. We found that neither film is stable under the considered gas environment, as their free energies of formation are +0.86 eV/$CoCO_3$ for 4 $CoCO_3$ on (3x3) Au(111) and +0.95 eV/$CoCO_3$ for 4 $CoCO_3$ on (2√3×2√3) Au(111).

1.7 Computed energetics of the reaction between CO and CoO_2/Au(111)

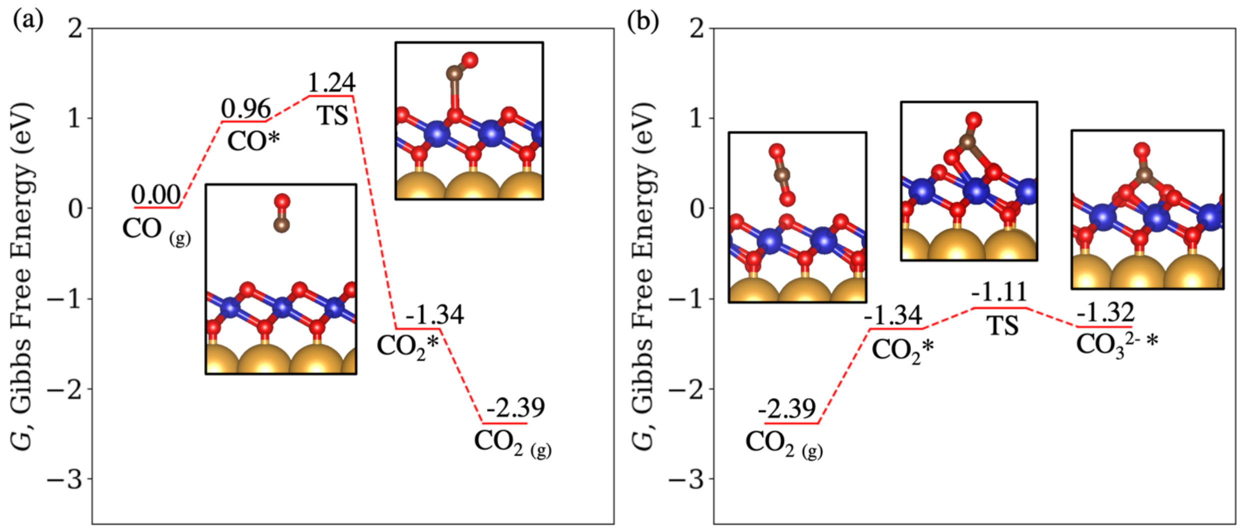

Figure S9: Free energy pathway of CoO_2/Au(111) reacting with gaseous CO and CO_2. CoO_2 reacts with CO gas by crossing a 1.24 eV free energy barrier, 0.2 eV higher than that for Co_3O_4. The formation of carbonates on CoO_2 is also unfavorable, as the carbonate decomposition barrier is low. The free energies of CO and CO_2 were calculated at 373.15 K, P_{CO} = 100 mTorr, and P_{CO2} = 0.1 mTorr.

In addition to Co_3O_4, a second $CoO_{x>1}$ model was also studied, which features an epitaxial CoO_2 trilayer (O-Co-O) film atop the Au(111) substrate (**Figure S9**). Unlike Co_3O_4/Au(111), CO cannot adsorb atop Co cations sandwiched between the O layers. Although only a 0.29 eV barrier is required for the reaction between physisorbed CO and lattice O, the entropy loss of CO gas is too high for this Eley-Rideal step to proceed as quickly as the surface reaction step over Co_3O_4. In fact, at 100 °C and a CO partial pressure of 100 mTorr, the net free energy barrier for CO gas-lattice O reaction is 1.24 eV, 0.2 eV higher than that over Co_3O_4. Like over Co_3O_4, weakly-bound CO_2 produced in this step can quickly react with lattice O by crossing a 0.23 eV barrier to form a bidentate CO_3^{2-} group. This carbonate group is rather unstable as only a 0.21 eV barrier is required to decompose it.

References

1. Powell, C.J. and A. Jablonski, *NIST Electron Inelastic-Mean-Free-Path Database 71, Version 1.0.* 1999.
2. Morgenstern, K., et al., *Cobalt growth on two related close-packed noble metal surfaces.* Surface Science, 2007. **601**(9): p. 1967-1972.
3. Weiss, W. and M. Ritter, *Metal oxide heteroepitaxy: Stranski-Krastanov growth for iron oxides on Pt(111).* Physical Review B, 1999. **59**(7): p. 5201-5213.

4. Walton, A.S., et al., *Interface controlled oxidation states in layered cobalt oxide nanoislands on gold.* ACS Nano, 2015. **9**(3): p. 2445-53.
5. Kersell, H., et al., *CO Oxidation Mechanisms on CoOx-Pt Thin Films.* J Am Chem Soc, 2020. **142**(18): p. 8312-8322.
6. Monkhorst, H.J. and J.D. Pack, *Special points for Brillouin-zone integrations.* Physical Review B, 1976. **13**(12): p. 5188-5192.
7. Giordano, L., et al., *Oxygen-Induced Transformations of an FeO(111) Film on Pt(111): A Combined DFT and STM Study.* The Journal of Physical Chemistry C, 2010. **114**(49): p. 21504-21509.PO
8. Giordano, L., et al., *Interplay between structural, magnetic, and electronic properties in aFeO ⁄ Pt(111)ultrathin film.* Physical Review B, 2007. **76**(7): p075416.
9. Allen, J.P. and G.W. Watson, *Occupation matrix control of d- and f-electron localisations using DFT + U.* Phys Chem Chem Phys, 2014. **16**(39): p. 21016-31.
